# Toward High-Performance Mg-Matrix Composites: Recent Advances in Ceramic Reinforcement Strategies and Processing Innovations

**DOI:** 10.3390/ma19020365

**Published:** 2026-01-16

**Authors:** Yuefeng Ying, Weideng Wang, Guoqiang You, Yan Yang, Bin Jiang, Lin Yue, Qilin Shao

**Affiliations:** 1 College of Materials Science and Engineering, Chongqing University, Chongqing 400044, China; 18105862520@163.com (Y.Y.);; 2 Chongqing Changan Automobile Company Limited, Chongqing 400023, China; 3 National Engineering Research Center for Magnesium Alloys, Chongqing University, Chongqing 400044, China; 4 National Key Laboratory of Advanced Casting Technologies, Chongqing University, Chongqing 400044, China

**Keywords:** magnesium matrix composites, ceramic particle reinforcement, mechanical properties, microstructure, fabrication processes, high-temperature performance, advanced lightweight materials

## Abstract

**Highlights:**

**What are the main findings?**

- Advances in ceramic particle types for Mg composites are summarized.
- Key preparation technologies and microstructures are compared.
- Strengths and limits of mainstream and emerging methods are clarified.

**What are the implications of the main findings?**

- Guides selection of reinforcements for high-performance Mg composites.
- Supports optimization of processing routes for improved properties.
- Identifies challenges and future directions for next-generation Mg materials.

**Abstract:**

Magnesium matrix composites formed by incorporating ceramic particles into a magnesium alloy matrix can effectively leverage the complementary properties of the matrix and reinforcement. This approach significantly enhances the mechanical properties of the material at both room and elevated temperatures, offering a viable solution to the inherent limitations of Mg alloys, such as insufficient absolute strength, stiffness, and poor heat resistance. This article reviews the latest research progress in the field of ceramic particle-reinforced magnesium matrix composites in recent years. First, the current research status of magnesium matrix composites reinforced with different types of ceramic particles is comprehensively summarized. Subsequently, it provides a summary and in-depth analysis of the principles, key technologies, and microstructural characteristics of both mainstream and emerging preparation processes, and discusses their advantages and disadvantages. Finally, the challenges in current research are analyzed, and future cutting-edge directions for developing high-performance ceramic particle-reinforced magnesium matrix composites are discussed.

## 1. Introduction

Magnesium alloys, which are the lightest metallic structural materials in practical use, are widely considered as ideal materials for aerospace, automotive, transportation, and biomedical applications [1,2,3,4,5,6,7,8] because their density is only two-thirds that of aluminum, one-fourth that of steel, and two-fifths that of titanium alloys. In addition, they offer advantageous characteristics such as high specific strength, good ductility, excellent damping capacity, and recyclability. However, the inherent weaknesses of magnesium alloys, such as their relatively low absolute strength, insufficient wear resistance, and poor high-temperature performance, severely limit their engineering applications in demanding environments such as high temperatures or heavy loads [9,10,11]. Moreover, magnesium exhibits poor formability at room temperature, which is primarily attributed to its crystal structure: the hexagonal close-packed (hcp) structure provides limited slip systems, resulting in insufficient plastic deformation capability at room temperature [12,13,14,15,16]. While modern industries demand stringent service conditions for structural materials, traditional Mg alloys fail to effectively fulfill such rigorous engineering requirements.

Magnesium matrix composites have been developed to overcome these limitations. By incorporating high-performance ceramic reinforcements into a pure magnesium or magnesium alloy matrix and leveraging the complementary properties of the matrix and reinforcement phases, the overall performance of the material can be significantly enhanced [17,18,19,20,21]. The addition of ceramic particles not only increases the strength and hardness of the matrix through dispersion strengthening, but also effectively inhibits grain-boundary sliding at high temperatures, thereby improving wear resistance and thermal stability. Commonly used reinforcements include carbides, borides, oxides, and nitrides, with their selection mainly based on physical and chemical compatibility with the Mg matrix [22,23,24,25,26]. Furthermore, mainstream manufacturing processes for ceramic-particle-reinforced magnesium matrix composites, such as casting and powder metallurgy, still face numerous challenges: reinforcements tend to agglomerate owing to interfacial energy differences, leading to uneven distribution; grain coarsening is prominent during casting; and controlling porosity in powder metallurgy is challenging. These defects hinder further enhancement of material properties [27,28,29].

The literature selected for this study is primarily sourced from the Web of Science database, focusing on publications related to magnesium-based composites from the past five years. Therefore, in this study, we systematically reviewed the latest research progress in the field of ceramic-particle-reinforced magnesium matrix composites. First, the influence of reinforcement selection and interface design on material properties was analyzed. Then, the breakthroughs achieved by novel manufacturing processes in addressing distribution uniformity and defect control were evaluated. Finally, this paper summarizes the key challenges in current research and outlines future technological pathways for developing composites with high service performance. This study aims to provide theoretical references and practical insights for the development of high-quality and high-efficiency materials within the field.

## 2. Magnesium-Based Composites with Single-Particle Reinforcements

Magnesium matrix composites (MMCs) are lightweight metallic materials fabricated by adding one or more reinforcement phases to a matrix of pure Mg or Mg alloys. The core characteristic of this material is the significant enhancement of its mechanical properties through the introduction of a reinforcement phase, giving it advantages such as low density, high specific strength, and high specific stiffness [30]. MMCs include those based on pure Mg [31] and Mg alloys [32,33,34]. Pure Mg offers a relatively high specific strength but suffers from insufficient absolute strength, and its strengthening via alloying has limited effectiveness. Conversely, the incorporation of high-hardness and high-stiffness reinforcements can effectively compensate for deficiencies inherent in the magnesium matrix. The ceramic particles commonly used in metal matrix composites include carbides, borides, oxides, and nitrides. These ceramic particles typically possess high hardness, high elastic modulus, good high-temperature resistance, and a low coefficient of thermal expansion. The addition of an appropriate amount of ceramic reinforcement can significantly enhance the material strength, hardness, and wear resistance through mechanisms such as load transfer, grain-refinement strengthening, and dislocation strengthening [35,36].

### 2.1. Carbides and Borides

Silicon carbide (SiC), a cost-effective ceramic strengthening phase, is widely used in magnesium-based composite materials. Studies have shown that its strengthening effect varies depending on the preparation process and associated challenges.

Studies on casting methods consistently confirm the grain refinement and strengthening/toughening effects of SiC. Khosravi et al. [37] fabricated SiC-particle-reinforced Mg matrix composites via stir casting. They found that 3 wt.% SiC drastically refined the composite grain size, resulting in significant improvements in ultimate tensile strength (UTS) and yield strength (YS). Furthermore, Sun et al. [38] demonstrated that using molds with high thermal conductivity for cooling effectively enhanced the flexural strength and fracture toughness of the composite. However, casting processes have inherent limitations. Kumar et al. [39] showed that although material hardness increased significantly with increasing SiC content, the composite density and porosity also increased. Additionally, Ahuir-Torres et al. [40] report that directly adding SiC particles to the AZ31 alloy increases its corrosion rate. The AZ31 casting sample, without ceramic particles, forms an effective passivation film, which can improve its corrosion resistance. However, adding ceramic particles to magnesium can impair corrosion resistance due to microgalvanic cell formation at the interfaces between the Mg matrix and second phase, thereby diminishing the chemical protection provided by the passive film.

**Figure 1:**
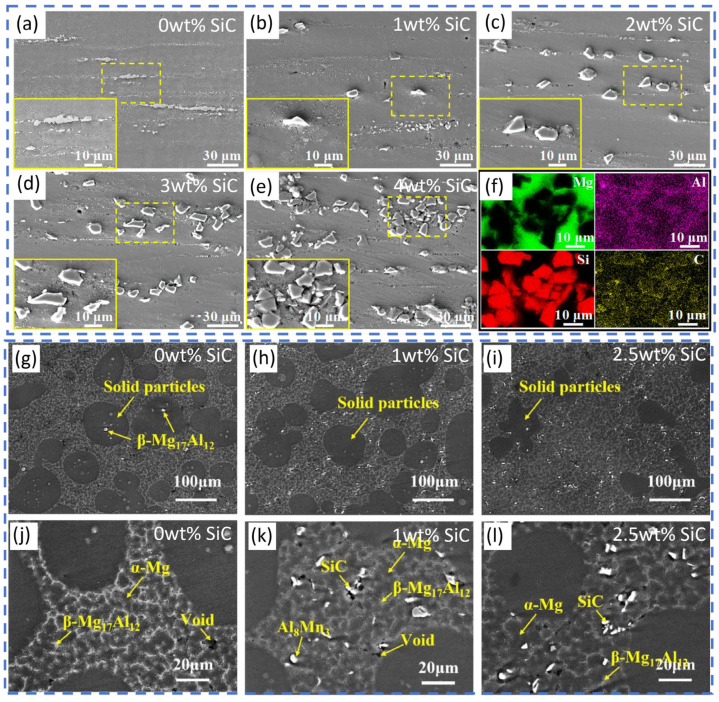
(**a**–**f**) Microstructures of SiC/AZ91D composites fabricated by powder metallurgy [41]; (**g**–**l**) microstructure of SiC/AZ91D composites produced via semi-solid injection molding [42].

Powder metallurgy offers a greater propensity for achieving uniform microstructures. An example is the SiC/AZ91D composite fabricated by Shi et al. [41]. In this composite, SiC particles were uniformly distributed along the grain boundaries (Figure 1a–f). This distribution not only refined the grains but also effectively suppressed crack initiation and propagation owing to strong particle–matrix bonding. Consequently, both YS and elongation (EL) increased simultaneously, demonstrating an excellent strength–ductility balance. Research has also revealed the unique interfacial characteristics and performance patterns of emerging processes such as semi-solid injection molding and additive manufacturing. Using semi-solid injection molding, Lu et al. [42] reported that although SiC particles were uniformly distributed and free from agglomeration (Figure 1g–l), minute voids existed adjacent to them. Moreover, in composites produced by this process, microhardness decreased with increasing SiC content, whereas tensile strength peaked at 2.5 wt.% SiC. This phenomenon is observed because the introduced reinforcement particles increase the defect density to some extent, and the composite’s final mechanical properties depend on the combined balance of the particles’ strengthening effect and the defects’ weakening effect. For extremely low or extremely high SiC contents, the detrimental impact of defects may outweigh the reinforcement effect, leading to performance degradation. Zhao et al. [43] investigated binder jet additive manufacturing and demonstrated that the introduction of SiC particles effectively refined the grains (refinement rate of 15.86%) and improved mechanical properties.

**Figure 2:**
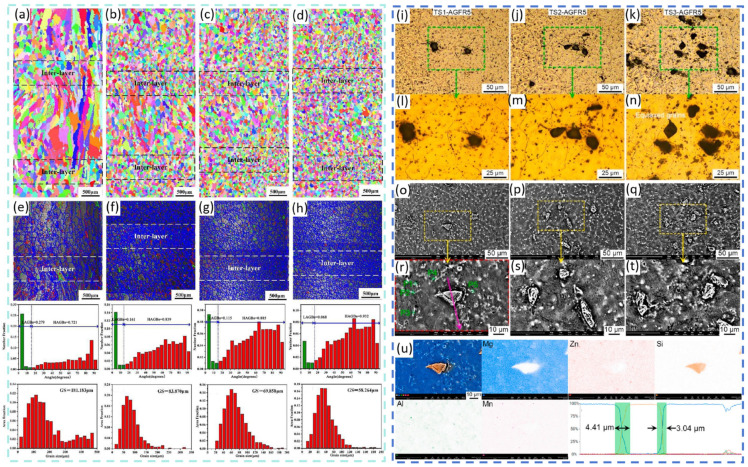
(**a**–**h**) Microstructures of SiC/AZ91D composites fabricated using interlayer coating combined with arc additive manufacturing technology [47]; (**i**–**u**) microstructure of SiC/AZ91D composites produced via powder-feeding with arc additive manufacturing technology [48].

Recently, researchers have employed wire-arc-directed energy deposition (WA-DED) to fabricate ceramic-particle-reinforced MMCs. This approach combines the performance advantages of composite materials with the unique characteristics of the WA-DED process, demonstrating broad development prospects [44,45,46]. Existing studies have shown that thin-walled magnesium matrix composite components produced via WA-DED possess fine microstructures and superior properties. Zhao et al. [47] introduced SiC particles as reinforcements during the WA-DED of AZ31B magnesium alloy using a layer-by-layer coating method. With the addition of SiC particles, the microstructure underwent significant transformation: the initial columnar grains evolved into coarse equiaxed grains and ultimately fine equiaxed grains. The average grain size decreased from 181.183 μm to 58.264 μm. As shown in Figure 2a–h, grain size in the interlayer regions was relatively small. This refinement is attributed to the added SiC particles acting as heterogeneous nucleation sites, which not only inhibited the formation of columnar grains but also effectively refined the weld grains. As a result, the mechanical properties of the components improved; compared with the unreinforced sample, both tensile strength and ductility increased. This strengthening originated from the increased number of grain boundaries (due to SiC-induced grain refinement), which hinder dislocation motion. Simultaneously, second-phase particles obstructed dislocations and contributed to load bearing/transfer. Thus, the mechanical performance of components deposited with SiC particles was optimized through the synergistic effect of multiple strengthening mechanisms. Zhang et al. [48] adopted a different approach by introducing SiC particles into an AZ31 magnesium alloy matrix using a coupled powder feeder for synchronous powder-feeding during WA-DED. The resulting composite component exhibited excellent internal quality, with no observed defects such as pores or cracks. As shown in Figure 2i–u, the addition of SiC promoted a transition from columnar to equiaxed grains, and increasing the SiC content further refined the equiaxed grains. Regarding mechanical properties, compared with the pure AZ31 matrix, the SiC-reinforced composite achieved maximum yield and tensile strengths of 92.8 MPa and 213.3 MPa, respectively. The maximum improvement in tensile strength reached 56%, whereas elongation did not change significantly, indicating that grain refinement played the primary strengthening role.

Zhu et al. [49] further developed a machine-learning prediction model based on existing datasets for SiC-reinforced MMCs. The model demonstrated excellent accuracy and reliability in predicting tensile strength. Analysis of key influencing factors indicated that both process parameters and material parameters significantly affect tensile strength. In summary, although SiC particles effectively enhance the strength and hardness of the magnesium matrix across different processes, their introduction presents common challenges such as increased porosity, difficulties in interfacial bonding control, and potentially worsened corrosion resistance.

**Figure 3:**
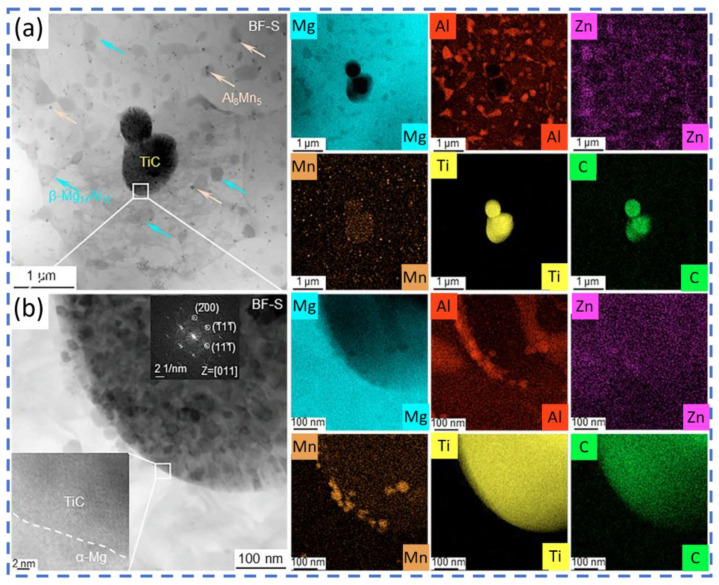
Microstructure of TiC/AZ91D composite material prepared by LPBF [52]. (**a**) Low-magnification and (**b**) high-magnification transmission electron microscopy images.

**Figure 4:**
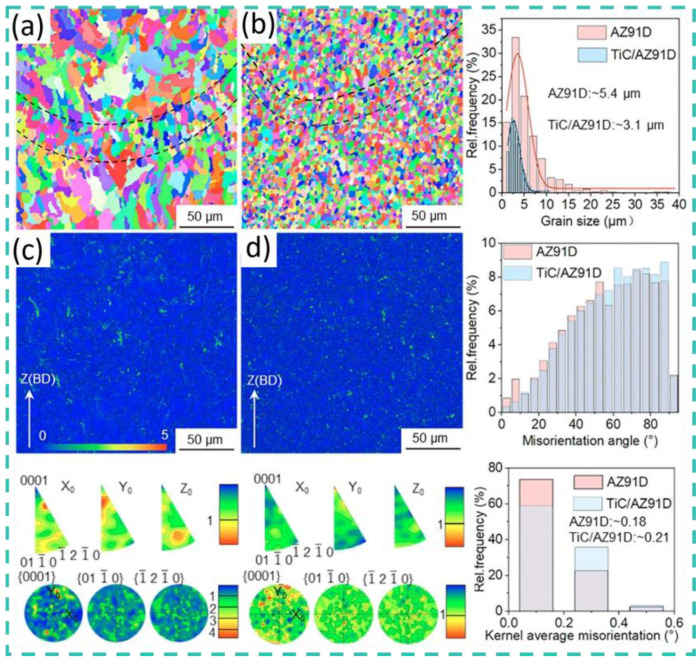
Microstructure of TiC/AZ91D composite material prepared by LPBF [52] (**a**,**b**) EBSD-IPF results (**c**,**d**) EBSD-KAM results.

Regarding other carbides, Rani et al. [50] fabricated a Ti_3_AlC_2_/AZ91 composite via melt infiltration. The composite exhibited high microhardness (307 HV), compressive strength (592 MPa), and flexural strength (560 MPa). Banerjee et al. [51] manufactured WC-particle-reinforced AZ31 MMCs using ultrasonically vibrated stir casting, which showed good wear resistance. They also analyzed the influence of various input parameters (load and speed) on output responses (wear loss and coefficient of friction), establishing a prediction model with minimal error. Xi et al. [52,53] produced a TiC/AZ91D magnesium matrix composite using laser powder bed fusion (LPBF). Its microstructure, shown in Figure 3 and Figure 4, demonstrated high hardness (114.6 HV), high tensile strength (345.0 MPa), and elongation (4.1%). This study shows that unmelted TiC particles within the composite effectively hinder crack propagation, thereby enhancing performance.

Among boride ceramic particles, B_4_C and ZrB_2_ demonstrate significant strengthening and toughening effects. Mitra et al. [54] fabricated a B_4_C/AZ91D composite using ultrasonically assisted stir casting and found that the addition of ceramic particles markedly enhanced the mechanical properties, resulting in optimal corrosion resistance at 1.5 wt.%. This study reveals that the introduction of B_4_C particles simultaneously enhances the corrosion resistance and mechanical properties of the composite. However, previous studies indicate that while introducing SiC particles improves mechanical properties, these particles may deteriorate the corrosion resistance. This result, to some extent, suggests that the relationship between corrosion resistance and mechanical properties in MMCs can be either positively or negatively correlated. Ultimately, the feasibility of achieving synergistic optimization of corrosion resistance and mechanical properties depends significantly on the reinforcing particles. Tasci et al. [55] employed powder metallurgy to produce a B_4_C/WE43 composite. The 2% B_4_C/WE43 composite exhibited the highest hardness and transverse rupture strength, along with the lowest wear rate. Ayyanar et al. [56] focused on process optimization and prepared a ZrB_2_/AZ91D composite via squeeze casting to reduce porosity and improve wear resistance; the composite’s mechanical properties surpassed those of similar stir-cast materials. Surendran et al. [57] used ultrasonically assisted stir squeeze casting to fabricate composites reinforced with TiC, TiB_2_, and TiN ceramic particles and demonstrated that the TiB_2_-reinforced composite exhibited the best tribological performance. Sahoo et al. [58] synthesized a TiB_2_-reinforced ZE41 composite in situ using stir casting, confirming that increasing TiB_2_ content improved both material strength and grain refinement; they also combined theoretical modeling and experiments to establish relationships among particle content, Young’s modulus, and YS, elucidating the strengthening mechanisms. Kelen et al. [59] found that although the ductility of TiNi-particle-reinforced composites decreased with increasing content, the reduction was less pronounced than that observed for other ceramic reinforcements, indicating potential advantages for strength–ductility matching.

### 2.2. Nitrides and Oxides

Several researchers focused on nitride reinforcement systems. Alam [60] and Dhinakarraj et al. [61] fabricated Si_3_N_4_ particle-reinforced MMCs via stir casting. Their studies consistently showed that adding Si_3_N_4_ improved microhardness and strength, though often at the cost of reduced ductility. López et al. [62] enhanced the wettability between Si_3_N_4_ particles and the magnesium matrix by oxidizing the particle surfaces to form an SiO_2_ layer, subsequently using this modified reinforcement to successfully prepare Si_3_N_4_-reinforced AZ91 composites through pressureless infiltration.

**Figure 5:**
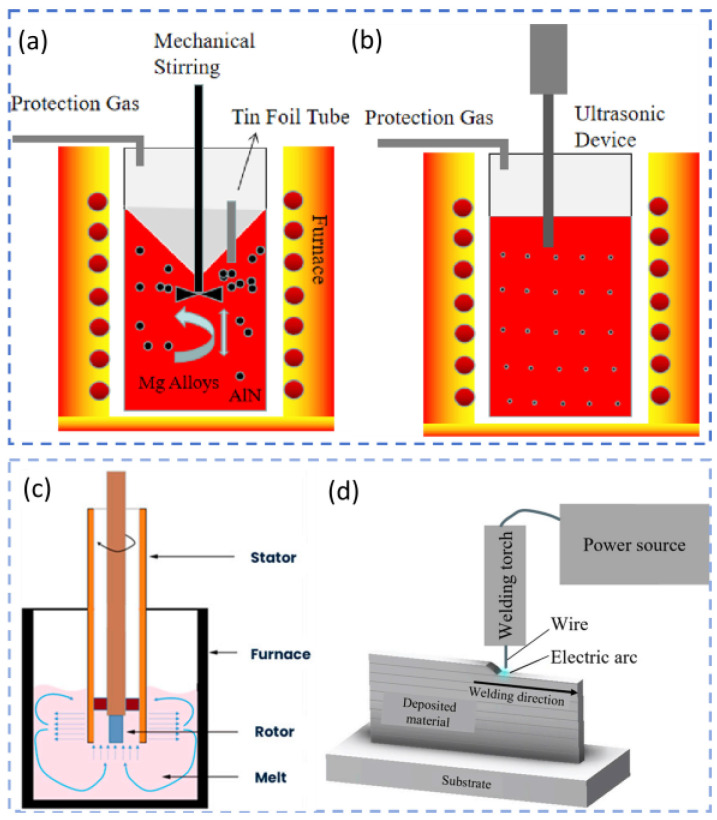
Different methods for introducing AlN ceramic particles: (**a**,**b**) External addition method [65]; (**c**,**d**) re-melting after compounding [66].

To overcome interfacial bonding challenges associated with externally added ceramic particles, researchers have also explored in situ synthesis routes. Aluminum nitride (AlN) is of particular interest because it can be introduced either by external addition or through in situ formation. Arreola-Fernández [63], Saboori [64], Wang [65], and Dieringa [66] effectively enhanced the mechanical, thermal, and electrical properties of magnesium composites by incorporating externally added AlN particles. Wang et al. further demonstrated that AlN nanoparticles not only refined matrix grains but also modified the morphology of the primary CaMgSn phase and suppressed grain coarsening, thereby significantly improving tensile properties and hardness at both room temperature and 250 °C. In contrast, the in situ synthesis route offers superior interfacial bonding. Dieringa et al. successfully introduced AlN particles into an AM60 alloy by fabricating composite welding wires reinforced with AlN nanoparticles (Figure 5c,d). The resulting composites exhibited refined grains, increased tensile strength, and a marked improvement in YS due to the combined effects of grain refinement, strengthening, and the Orowan mechanism.

Reddy et al. [67] achieved in situ formation of AlN particles by injecting ammonia gas into a Mg–Al alloy melt at 1173 K for 70 min. They observed that AlN yield increased with higher ammonia flow rates, with the generated particles enveloped by the γ-(Mg,Al) phase. Experimental findings closely matched thermodynamic predictions, confirming that the reaction proceeds near equilibrium.

**Figure 6:**
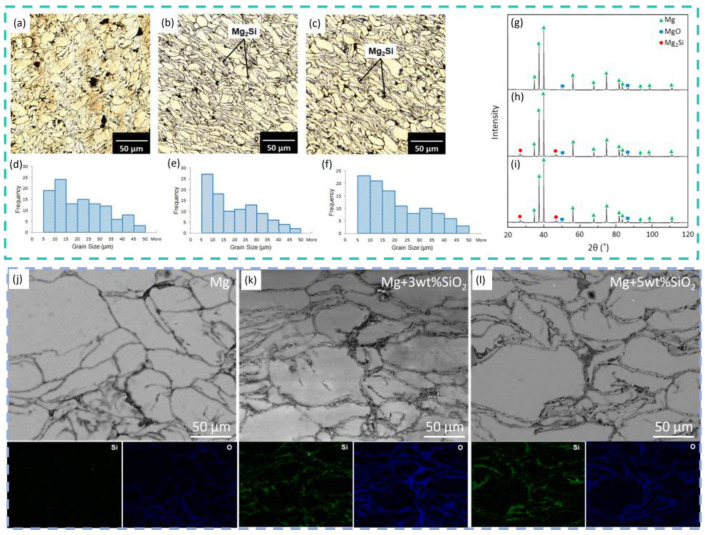
(**a**–**l**) Microstructures of SiO_2_/AZ31 composites fabricated by powder metallurgy [69].

For oxide-based reinforcement systems, Babu et al. [68] reported that increasing La_2_O_3_ content raised the hardness of their fabricated La_2_O_3_/AZ31 composites, while 1.5 wt.% La_2_O_3_ yielded the lowest coefficient of friction (0.20) and the smoothest wear surface. Iqbal et al. [69] fabricated nano-SiO_2_-reinforced magnesium composites (Figure 6a–l) and found that SiO_2_ significantly improved hardness and reduced degradation rates in simulated physiological environments, thereby enhancing corrosion resistance. Paul et al. [70] developed SrTiO_3_-reinforced composites that exhibited bimproved corrosion resistance as well as non-toxicity and mild antibacterial behavior. Ercetin et al. [71], studying Al_2_O_3_/Mg–Zn composites, determined that 8 wt.% Al_2_O_3_ maximized tensile strength, whereas 2 wt.% provided the best corrosion resistance. Bassiouny et al. [72] produced a graded Y_2_O_3_-reinforced magnesium composite using centrifugal casting. The outer layer, with higher reinforcement content and density, displayed superior mechanical properties, including a hardness of 106.89 HV, tensile strength of 197.39 MPa, and compressive strength of 335.29 MPa. Parande et al. [73] synthesized Mg composites reinforced with micron-, submicron-, and nano-sized CeO_2_ particles using disintegrated melt deposition and observed that smaller particle sizes enhanced both mechanical and thermal properties, particularly compressive behavior. Vinitha et al. [74] reported that Ca_3_(PO_4_)_2_ particles improved the mechanical performance of AZ91D, while Mohanavel et al. [75] confirmed that ZrSiO_4_/AZ91 composites possessed excellent mechanical and wear resistance properties. Within powder-metallurgy-based fabrication routes, various reinforcements also offer distinct advantages. Saleh et al. [76] incorporated squid quill ash (SQA) particles (mainly CaCO_3_) into AZ91 via stir casting, producing a low-cost composite. With 10 wt.% SQA, the hardness, YS, and UTS increased by 32.34%, 18.86%, and 12.34%, respectively, compared with those of the base alloy. In contrast, the wear resistance improved by 14.05% and 30.81% for 5 and 10 wt.% SQA addition, respectively. These findings demonstrate the feasibility of using SQA particles in the production of MMCs. This method shows comparatively less negative environmental impact by repurposing SQA as well as offers a practical solution for the economical utilization of SQA particles in the fabrication of MMCs for aerospace and automotive applications.

Ghazizadeh et al. [77] produced hydroxyapatite (HA)-reinforced composites via stir casting followed by extrusion, noting that HA refined the microstructure but increased porosity; nevertheless, HA enhanced corrosion resistance, with Mg/2.5 wt.% HA showing the greatest benefit. Similarly, Kasaeian-Naeini et al. [78] fabricated HA-reinforced composites via stir casting followed by Equal Channel Angular Pressing (ECAP). HA slightly improved fracture toughness, while subsequent ECAP treatment increased toughness more substantially, ultimately reaching 27.62 MPa·m^0^·^5^.

In summary, a wide range of ceramic reinforcement phases—including carbides, borides, nitrides, and oxides—have been successfully demonstrated to enhance the mechanical, wear, and corrosion properties of MMCs produced through various manufacturing routes. As research progresses, increasing attention is being given to combining multiple reinforcement types to achieve synergistic strengthening.

## 3. Magnesium-Based Composites with Multiple Particle Reinforcements

**Figure 7:**
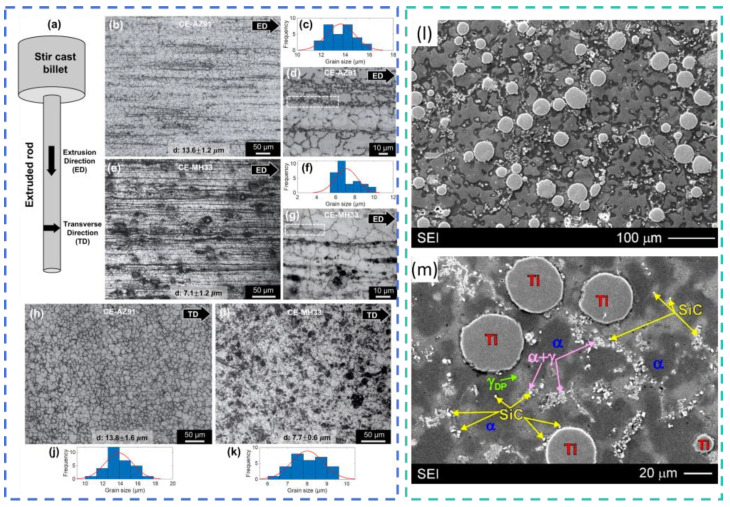
(**a**–**k**) Microstructure of (SiC + Fly Ash)/AZ91 composite material [81]. (**l**,**m**) Microstructure of (SiC + Ti)/AZ91 composite material [82].

Casting and its derivative processes remain the primary methods for fabricating ceramic-particle-reinforced MMCs. Raj et al. [79] produced SiC- and BN-reinforced AZ91D composites via stir casting and observed uniform particle dispersion, refined grains, and strong interfacial bonding, which collectively enhanced wear resistance. Vaziri et al. [80], also using stir casting, prepared (ZrB_2_ + SiC)/AZ31 composites and found that the 1.5 vol.% ZrB_2_ − 0.5 vol.% SiC ratio resulted in the finest grains and the most favorable creep behavior. Gollapalli et al. [81] fabricated AZ91 composites reinforced with 3 vol.% SiC and Fly Ash (Figure 7a–k); after hot extrusion at 325 °C, the grain size was refined from 13.6 μm to 7.1 μm, Vickers hardness increased from 73 HV to 111 HV. Braszczyńska-Malik et al. [82] successfully manufactured (SiC + Ti)/AZ91 composites via stir casting (Figure 7l,m); Ti particles were uniformly dispersed, while SiC particles were located within α-phase dendrites, on Ti particle surfaces, and in interdendritic eutectic regions, leading to improved mechanical properties relative to the unreinforced alloy. Gotagunaki et al. [83] prepared (Y_2_O_3_ + CeO_2_)/AZ91D composites using stir casting and achieved a 9–29% enhancement in wear resistance. Verma et al. [84] developed (HA + Al_2_O_3_@TiO_2_)/Mg composites via stir casting; these reinforcements improved mechanical strength and corrosion resistance, while the composites also demonstrated good biocompatibility, indicating suitability for orthopedic implants.

Gnanavelbabu et al. [85] fabricated (TiN + BN)/AZ91D composites through ultrasonically assisted casting and found that the reinforcement particles enhanced both corrosion and wear resistance. Zhu et al. [86] used ultrasonically vibrated semi-solid stir casting to prepare (SiC + TiC)/AZ91 composites with a uniform nanoparticle distribution; increasing nanoparticle content improved tensile strength and microhardness, and extrusion further refined matrix grains, particularly at higher reinforcement levels. Krishnan et al. [87] synthesized (SiC + BN)/Mg–Zn composites via ultrasonically vibrated stir casting and recorded enhanced corrosion resistance and a 41% improvement in fatigue strength compared with the matrix alloy. Krishnakumar et al. [88] employed argon-shielded stir casting to fabricate SiC- and BF-reinforced composites, determining that 6 wt.% SiC + 9 wt.% BF produced low porosity (1.03%), high tensile strength (359 MPa), and a low volumetric wear rate (5.36 mm^3^/m). Srilatha et al. [89] produced SiC- and B_4_C-reinforced magnesium composites via vacuum-assisted stir casting, with the 3 wt.% SiC + 2 wt.% B_4_C formulation yielding the highest compressive strength and microhardness. Sharma et al. [90] developed AZ91D composites reinforced with recycled waste glass powder and Si_3_N_4_ via vacuum stir casting; the addition of these reinforcements significantly improved tensile properties and wear resistance. Gu et al. [91] fabricated (SiC + Al_2_O_3_)/Mg-10Zn composites via thixoforming and achieved a YS of 186 MPa, an elastic modulus of 48.6 GPa, and 6.8% elongation. The reinforcement particles hindered the formation of large shear bands and improved ductility. The microstructure shows microcracks within SiC particles while the SiC/Mg interface remained intact, whereas partial fracture occurred at some Al_2_O_3_/Mg interfaces, demonstrating the load-bearing function of the reinforcement.

**Figure 8:**
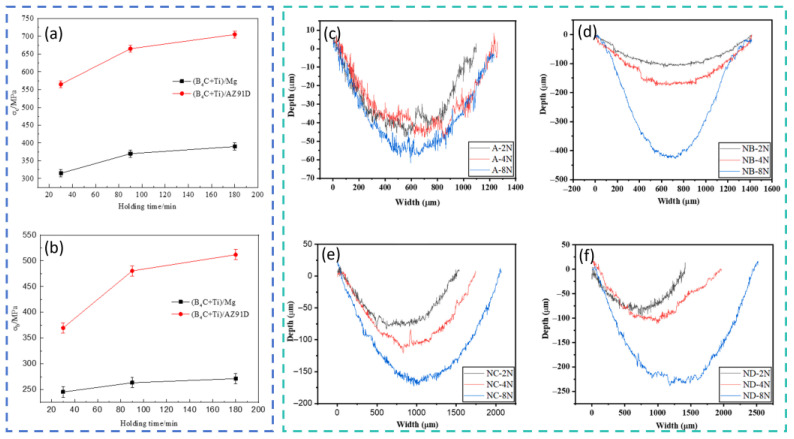
Mechanical properties of (B_4_C + Ti)/Mg and (B_4_C + Ti)/AZ91 composites [94] (**a**) compressive strength (**b**) bending strength; (**c**–**f**) mechanical Properties of (SiC + Ti)/Mg composite materials [95].

In situ techniques and powder metallurgy methods offer notable advantages for improving interfacial bonding and achieving uniform particle dispersion, making them well suited for producing high-performance magnesium composites. Zhang et al. [92] synthesized MgO and Al_3_Fe particles in situ through the reaction between AZ91 and Fe_3_O_4_, obtaining composites with fewer and smaller precipitates and refined grains. After extrusion, the composites exhibited YS, UTS, and elongation values of approximately 415.4 MPa, 480.1 MPa, and 5.9%, respectively. Zhang et al. [93] proposed a method involving the in situ synthesis of GNPs and MgO nanoparticles combined with SiC reinforcement; the (GNPs + MgO) phases helped transfer load during deformation and reduced interfacial stress concentration at SiCp, resulting in a ~70% increase in ductility and a 10% increase in modulus compared with SiCp/Mg-6Zn. Chen et al. [94] employed in situ reactive infiltration to prepare (B_4_C + Ti)/AZ91D composites, which displayed significantly improved compressive properties (Figure 8a,b). Ahmadian et al. [95] fabricated Mg composites reinforced with Ti and SiC via powder metallurgy, achieving substantial improvements in wear resistance (Figure 8c–f). Kumar et al. [96] employed the casting technique to prepare Mg composites reinforced with hybrid (1.5 wt.% Al_2_O_3_ + x wt.%TiB_2_) particles (x = 1, 3, 5). The hybrid reinforcements reduced grain size and transformed the β-Mg_17_Al_12_ phase into an acicular morphology, with optimal performance at 3 wt.% TiB_2_. Macit et al. [97] produced AZ91 composites reinforced with (ZnO + BN) nanoparticles via powder metallurgy; the AZ91-ZnO-10BN composite exhibited higher hardness (85 HB), improved wear resistance, a lower friction coefficient, and its wear loss was successfully modeled using a hybrid genetic algorithm–support vector regression method (98.80% accuracy). Fahad et al. [98] prepared (WC + SiO_2_)/AZ91D composites by powder metallurgy and achieved significant hardness improvements; the wear rate increased with load but decreased with sliding speed due to the formation of a lubricating layer, reaching a wear rate of only 0.0017 mm^3^/m and a friction coefficient of 0.33 under 10 N load and 150 mm/s speed. Mustu et al. [99] fabricated TiB_2_/ZK60 and (TiB_2_ + GNPs)/ZK60 composites via powder metallurgy; TiB_2_ was uniformly dispersed, whereas GNPs showed agglomeration. The 15 wt.% TiB_2_ composite exhibited the best wear behavior at 10 and 40 N loads and achieved the highest hardness (87.1 HV), compressive YS (290.1 MPa), and ultimate compressive strength (379.2 MPa). Moustafa et al. [100] produced (Fly Ash + TiO_2_/BN/B_4_C)-reinforced Mg composites by powder metallurgy; the reinforcements significantly enhanced mechanical properties. Fayomi et al. [101], using powder metallurgy at 500 °C, 30 MPa, 5 min holding time, and a 75 °C/min heating rate, fabricated Mg composites reinforced with 4 wt.% AlN + 4 wt.% Y_2_O_3_ + 4 wt.% VB, obtaining high-density materials. Their results indicated that composite density increased with temperature.

Table 1 summarizes the tensile properties of ceramic-particle-reinforced MMCs. Evidently, the ductility of multi-particle-reinforced composites is generally lower than that of reinforced composites with a single ceramic phase. However, the former often demonstrate superior wear resistance. The distinct microstructures resulting from single- and multi-particle reinforcements lead to substantial differences in the performance of magnesium composites. Thus, depending on specific engineering requirements, composites with tailored properties can be designed. Unlike MMCs fabricated using conventional casting methods, the processes reported in recent years have enabled breakthroughs in manufacturing high-performance composites. As shown in Table 1, wire-arc-directed energy deposition and LPBF can produce MMCs with excellent strength and plasticity likely owing to the rapid solidification rates of these processes, which refine the microstructure and thereby enhance the mechanical properties.

## 4. Heat Treatment of Magnesium-Based Composite Materials

**Figure 9:**
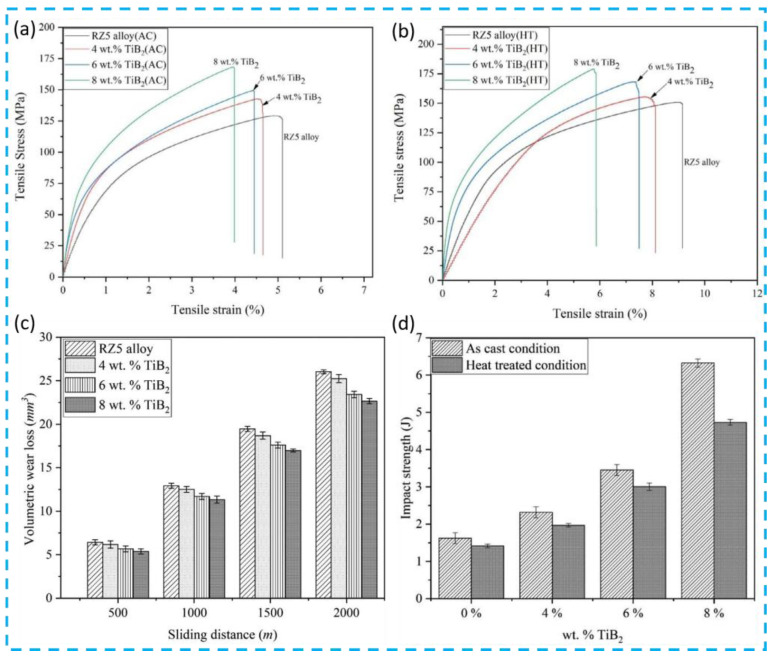
(**a**–**d**) Mechanical properties of as-cast TiB2/RZ5 composites before and after heat treatment [104].

Huang et al. [102] subjected SiC/AZ61 magnesium alloys to solution + aging treatment process. After treatment, the Mg_17_Al_12_ phase dissolved and reprecipitated. Their results indicated that the SiC particle content significantly influenced the post-treatment phase composition: an excessively high SiC fraction promoted the formation of brittle phases, thereby degrading the mechanical performance of the composites. Chang et al. [103] conducted solution + aging treatment on extruded SiC/AZ61 alloys. They reported that precipitates preferentially formed at SiCp/AZ91 interfaces and along grain boundaries near small SiC particles within extrusion bands; subsequently, these precipitates coarsened and extended into particle-free regions. Ahuir-Torres et al. [40] further demonstrated that heat treatment dissolves intermetallic phases such as β-Mg_17_Al_12_ and Al_8_Mn_5_, thereby improving the corrosion resistance of heat-treated SiCp/AZ31 composites. Meher et al. [104] fabricated TiB_2_/RZ5 composites and evaluated the mechanical properties of as-cast alloys, as-cast composites, heat-treated alloys, and heat-treated composites. Their results, shown in Figure 9a–d, revealed that heat treatment enhanced both hardness and tensile properties. Zhao et al. [105] investigated the microstructural evolution and interfacial bonding characteristics of GNPs/AZ91 composites subjected to T4, T5, and T6 heat treatments. Key findings include: during T4 treatment, the β-phase in the composite progressively decomposed and dissolved into the α-phase matrix, while excessive solution time led to microstructural overburning due to overheating. T5 treatment resulted in extensive β-Mg_17_Al_12_ precipitation with increasing aging time. During T6 treatment, numerous finely dispersed β-Mg_17_Al_12_ particles precipitated within the composite, providing significant strengthening. Moreover, T6 treatment facilitated the diffusion of GNPs into the matrix and grains. Acting as heterogeneous nucleation sites for precipitates, GNPs promoted second-phase precipitation and contributed to forming a tightly interlocked three-dimensional structure at the GNP/Mg interface, markedly enhancing interfacial bonding strength. Liu et al. [106] applied semi-solid isothermal heat treatments at various temperatures to Fly Ash/AZ91 composites and observed improvements in damping capacity.

Sharma et al. [107] prepared (SiC + TiO_2_)/Mg–Al–Zn composites and subjected them to solution + aging treatment. Their microstructural observations revealed that at lower aging temperatures, Mg_17_Al_12_ precipitates formed primarily through grain-boundary diffusion, resulting in discontinuous boundary precipitates. At higher temperatures, bulk diffusion became dominant, enabling Mg_17_Al_12_ precipitation both at boundaries and within grains. Additionally, Mg_17_Al_12_ phases formed around SiC particles, indicating that SiC acted as heterogeneous nucleation sites. Sahoo et al. [108] fabricated (TiC + TiB_2_)/AZ91 composites and performed solution + aging treatment, showing that the introduction of (TiC + TiB_2_) accelerated aging-precipitation kinetics. This was attributed to an increased interfacial area that provided abundant nucleation sites, enhanced solute diffusion, and elevated dislocation density that offered additional pathways for nucleation and growth. Gnanavelbabu et al. [85] conducted T6 heat treatments at different aging temperatures on (TiN + BN)/AZ91D composites and found that samples aged at 200 °C exhibited optimal corrosion resistance (Ecorr = −1.38 V, I_corr_ = 1.82 × 10^−6^ A/cm^2^).

Overall, reinforcement particles strongly influence recrystallization and precipitation during heat treatment. They hinder dislocation motion, leading to the accumulation of high-density dislocations with pronounced orientation gradients. These dislocations rearrange to form subgrain boundaries, thereby promoting dynamic recrystallization [21]. Reinforcement particles also pin grain boundaries and suppress the growth of recrystallized grains. Jiang et al. [109] reported that, after solution and aging treatments, ceramic-particle-reinforced magnesium composites exhibited enhanced recrystallization and retained stable recrystallized microstructures. Additionally, mismatch in the coefficient of thermal expansion between reinforcement particles and the matrix increases dislocation density and stored deformation energy within the composites, thereby elevating the nucleation driving force and refining precipitates [110]. Reinforcement particles may also serve directly as heterogeneous nucleation sites, further promoting precipitate refinement [103].

As with traditional magnesium alloys, the performance of ceramic particle-reinforced MMCs can be improved through process optimization [14,111] and composition optimization [112,113]. However, unlike in the case of traditional alloys, the presence of ceramic particles can significantly affect the microstructure and performance, and is thus an important area of research focus.

**Figure 10:**
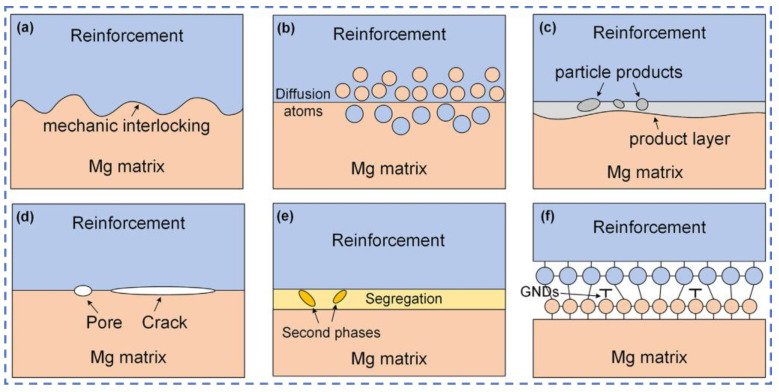
Schematic diagram of the interfacial structures caused by various interactions [114]: (**a**) mechanic interlocking; (**b**) elements inter-diffusion; (**c**) interfacial chemical reaction; (**d**) interfacial defects; (**e**) interfacial segregation of alloying elements; (**f**) interfacial GNDs.

Heat treatment affects, to some extent, the interfacial bonding between ceramic particles and the magnesium alloy matrix. The bonding between ceramic particles and the magnesium alloy matrix involves four mechanisms (Figure 10): (i) mechanical interaction, (ii) physical wetting interaction, (iii) chemical interaction, and (iv) substructure formation at the interface [114]. (i) Mechanical interactions: These interactions are induced through mechanical interlocking or via a combination of interlocking and sliding friction at the interface. (ii) Physical wetting interactions: during bonding enhancement in magnesium, atoms near the interface are attracted by electrostatic or interatomic (intermolecular) forces to form interfacial bonds; this physical attraction constitutes a significant interfacial bonding interaction, particularly for smooth interfaces lacking chemical interactions. (iii) Chemical interactions: The interface in MMCs is susceptible to interfacial atomic diffusion and chemical reactions, which result in a more complex structure with stronger interfacial bonds. (iv) Substructure at the interface: Beyond the direct interaction between the reinforcement and matrix, certain substructures, such as high-density dislocations and interfacial precipitates, are often generated within the interfacial region. The strength of the bonding force between the ceramic particles and matrix is reflected by the interfacial bonding energy. The more negative the bonding energy between particles, the higher the energy required to separate them, and consequently, the stronger their bonding force [115]. After heat treatment, the phases at the interface may change, which can affect the aforementioned interaction mechanisms and thereby influence the interfacial bonding performance.

## 5. Engineering Applications of Magnesium-Based Composites

**Figure 11:**
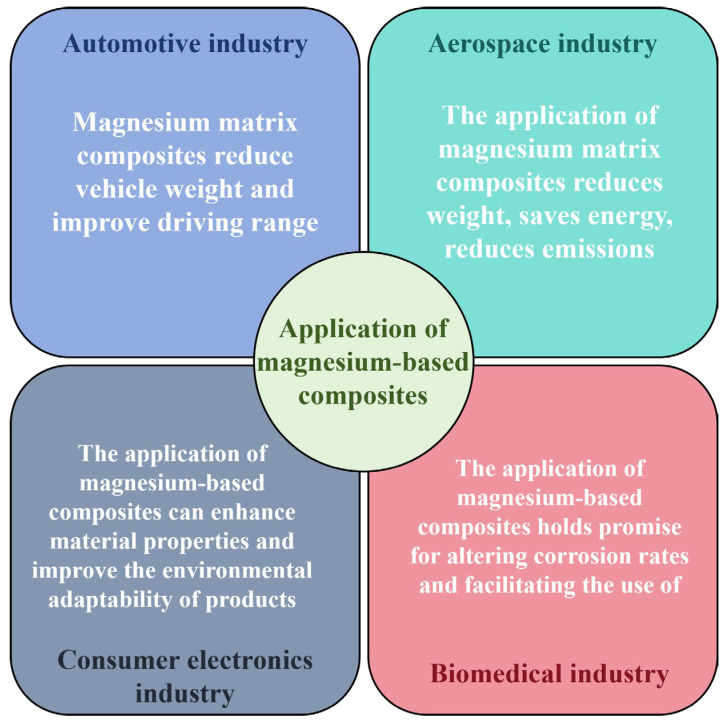
Application fields and advantages of magnesium-based composites.

In recent years, with the optimization of preparation processes and the enhancement of properties, magnesium matrix composites are considered a potential alternative to traditional magnesium alloys and composites in aerospace, automotive, electronics, and biomedical fields [116]. The application fields and advantages of magnesium-based composites are illustrated in Figure 11. In the automotive industry, weight reduction can lower energy consumption; replacing current materials with magnesium and its composites can effectively reduce weight while maintaining equivalent mechanical properties. Especially with the development of new energy vehicles, reducing weight can significantly improve the key performance indicator of driving range. The aerospace industry similarly has weight reduction demands, and magnesium matrix composites can reduce fuel consumption. Furthermore, compared to traditional magnesium alloys, the heat resistance of magnesium matrix composites has been significantly improved, which can further promote their application in the aerospace sector. In the consumer electronics industry, electromagnetic shielding capability and resistance to environmental or outdoor temperatures are key requirements. Magnesium possesses excellent electromagnetic shielding properties, and magnesium matrix composites can maintain this advantage while providing good mechanical properties to handle complex outdoor environments. In the biomedical field, magnesium matrix nanocomposites also hold application potential. Magnesium is abundant in the human body and can serve as an implant material. Currently, many researchers are studying the corrosion resistance of magnesium matrix composites [69,70]. Developing novel magnesium matrix composites with controllable corrosion rates is expected to promote the advancement of magnesium alloys in the medical field.

## 6. Conclusions and Outlook

**Figure 12:**
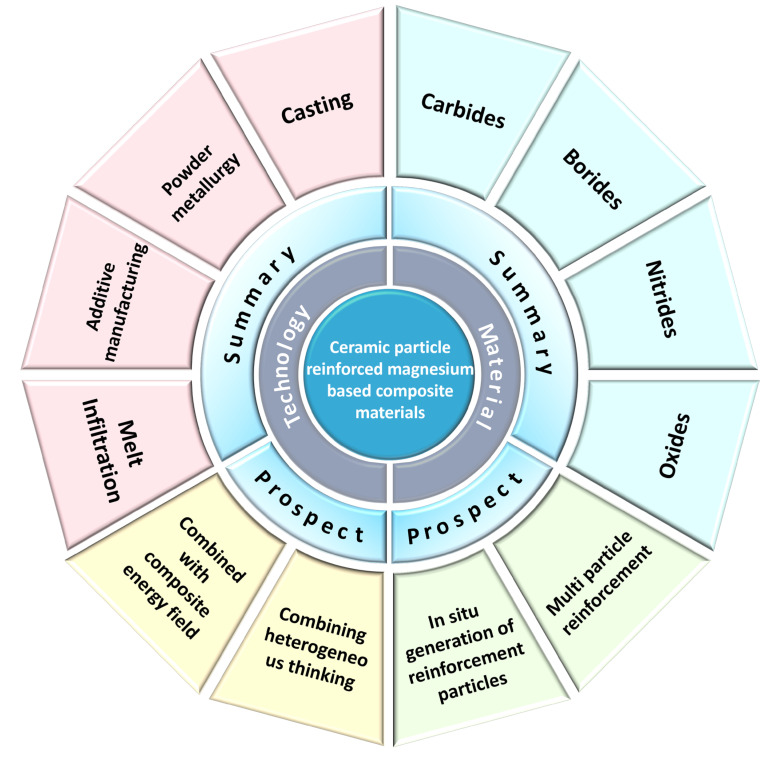
Overview of current research status and future prospects for ceramic-particle-reinforced magnesium-based composites.

In summary, ceramic particle-reinforced MMCs exhibit substantial potential for the development of high-performance structural components. Although significant progress has been made, research in this area remains in a developmental phase, leaving ample room for further exploration and broader application, as illustrated in Figure 12. Based on current advancements, future work should prioritize the following four key directions to drive the efficient and high-quality development of these composites:

(1) Systematic investigation of diverse ceramic reinforcements: future studies should explore MMCs reinforced with a wide range of ceramic particles, focusing on their effects and strengthening mechanisms. Establishing a comprehensive process–structure–property database will support the rational design and control of microstructures and enable the optimization of composite performance.

(2) Design of novel reinforcement systems with improved interfacial compatibility: for MMCs, interfacial control critically influences their properties. Effective interfacial bonding can significantly improve the reinforcement particle distribution and defect characteristics, which in turn enhance the mechanical performance. Therefore, developing new reinforcement formulations—that are tailored for magnesium alloys and enhance the interfacial bonding while synergistically optimizing the strength–ductility balance of the composites—is crucial.

(3) Optimization of fabrication processes and microstructure regulation: improving reinforcement incorporation techniques, refining matrix microstructures, strengthening interfacial bonding, and ensuring uniform reinforcement distribution are essential for achieving superior mechanical properties. Advanced processing strategies should therefore be systematically refined and integrated.

(4) Integration of advanced concepts and hybrid processing technologies: future efforts should incorporate innovative approaches—such as hybrid energy fields (ultrasonic, magnetic, or electromagnetic) for microstructure regulation, heterogeneous design concepts, and layer-wise strengthening via wire-arc-directed energy deposition. These strategies offer promising pathways for targeted reinforcement and the development of hierarchical or functionally graded composite structures.

## Figures and Tables

**Table 1:** Tensile properties of ceramic-particle-reinforced MMCs.

Material	Technology	YS (MPa)	UTS (MPa)	EL (%)	References
AZ91 + 2 wt.% SiC	Casting	73 ± 1.6	114 ± 3.4	10 ± 1.3	[39]
AZ91 + 5 wt.% SiC	Casting	64 ± 1.2	138 ± 2.6	8 ± 0.9	[39]
AZ91 + 8 wt.% SiC	Casting	97 ± 1.4	141 ± 2.7	12 ± 1.2	[39]
AZ91 + 11 wt.% SiC	Casting	126 ± 2.0	196 ± 3.8	7 ± 1.4	[39]
AZ91 + 3 wt.% SiC	Hot extrusion after sintering	172 ± 8	341 ± 11	4.43 ± 0.18	[41]
AZ91 + 1 wt.% SiC	Binder jetting	/	~172	~4.2	[43]
AZ31 + 2 wt.% SiC	Wire-arc-directed energy deposition	/	~233.35	~24.04	[47]
AZ91 + 2 wt.% TiC	Laser powder bed fused	/	~345	~4.1	[52]
ZE41 + 5 wt.% TiB_2_	Casting	37 ± 4	172 ± 5	8.2 ± 0.4	[58]
ZE41 + 10 wt.% TiB_2_	Casting	62 ± 2	224 ± 4	5.3 ± 0.3	[58]
ZE41 + 15 wt.% TiB_2_	Casting	56 ± 5	191 ± 7	4.0 ± 0.7	[58]
AZ31 + 2 wt.% Si_3_N_4_	Casting	/	~255	~15.9	[61]
AZ31 + 4 wt.% Si_3_N_4_	Casting	/	~257	~15.6	[61]
AZ31 + 6 wt.% Si_3_N_4_	Casting	/	~271	~14.7	[61]
AZ31 + 8 wt.% Si_3_N_4_	Casting	/	~283	~14.4	[61]
AZ31 + 10 wt.% Si_3_N_4_	Casting	/	~292	~13.9	[61]
TX31 + 0.1 wt.% AlN	Casting	~68.5	~137.1	~3.7	[65]
TX31 + 0.25 wt.% AlN	Casting	~78.7	~143.8	~3.3	[65]
TX31 + 0.5 wt.% AlN	Casting	~82.3	~160.8	3.8	[65]
TX31 + 1 wt.% AlN	Casting	~81.8	~147.5	3.0	[65]
AM60 − 1.5CAa + 1 wt.% AlN	Wire-arc-directed energy deposition	131 ± 5	226 ± 6	4.3 ± 0.8	[66]
Mg_2_Zn + 2 wt.% Al_2_O_3_	Casting	/	~160	~9.8	[71]
Mg_2_Zn + 8 wt.% Al_2_O_3_	Casting	/	~191	~4.4	[71]
AZ91 + 5 wt.% SQA	Casting	135.4 ± 2.5	178.7 ± 3	3.92 ± 0.3	[76]
AZ91 + 10 wt.% SQA	Casting	146.8 ± 3	189.3 ± 5	3.16 ± 0.25	[76]
AZ91 + (7.5 vol.% SiC + 7.5 vol.% Ti)	Casting	~118	~141	/	[82]
AZ91 + (1 wt.% Y_2_O_3_ + 1 wt.% CeO_2_)	Casting	~129.5	~155.41	~3.13	[83]
AZ91 + (2 wt.% Y_2_O_3_ + 1 wt.% CeO_2_)	Casting	~149.18	~179.02	~2.57	[83]
AZ91 + (3 wt.% Y_2_O_3_ + 1 wt.% CeO_2_)	Casting	~172.68	~207.22	~2.15	[83]
Mg + (1.2 wt.% HA + 0.7 wt.% Al_2_O_3_)	Casting	32.03 ± 3.47	87.18 ± 14.21	11.19 ± 3.47	[84]
Mg + (1.2 wt.% HA + 0.7 wt.% TiO_2_@Al_2_O_3_)	Casting	31.26 ± 2.15	85.85 ± 6.85	9.1 ± 0.48	[84]
AZ91 + (0.25 wt.% SiC + 0.25 wt.% TiC)	Casting	~287.3	~346.3	~3.4	[86]
AZ91 + (0.5 wt.% SiC + 0.5 wt.% TiC)	Casting	~314.6	~396.0	~5.8	[86]
AZ80 + (6 wt.% SiC + 3 wt.% BF)	Casting	274 ± 3	345 ± 3	2.114 ± 0.03	[88]
AZ80 + (6 wt.% SiC + 6 wt.% BF)	Casting	281 ± 3	351 ± 4	2.154 ± 0.02	[88]
AZ80 + (6 wt.% SiC + 9 wt.% BF)	Casting	285 ± 6	359 ± 7	2.114 ± 0.01	[88]
Mg + (2 wt.% SiC + 3 wt.% B_4_C)	Casting	~124.7	~156.9	/	[89]
Mg + (3 wt.% SiC + 2 wt.% B_4_C)	Casting	~119.5	~162.6	/	[89]
Mg + (2.5 wt.% SiC + 2.5 wt.% B_4_C)	Casting	~125.4	~173.5	/	[89]
AZ91 + (1.5 wt.% Al_2_O_3_ + 1 wt.% TiB_2_)	Casting	~73.62	~119.16	~1.183	[96]
AZ91 + (1.5 wt.% Al_2_O_3_ + 3 wt.% TiB_2_)	Casting	~108.8	~176.21	~1.006	[96]
AZ91 + (1.5 wt.% Al_2_O_3_ + 5 wt.% TiB_2_)	Casting	~89.5	~144.84	~0.96	[96]

## Data Availability

No new data were created or analyzed in this study. Data sharing is not applicable to this article.
